# Validating medical digital twins for clinical decision support: beyond predictive accuracy

**DOI:** 10.1093/jamiaopen/ooag160

**Published:** 2026-08-04

**Authors:** Alexandre Vallée

**Affiliations:** Department of Epidemiology and Public Health, Foch Hospital, Suresnes 92150, France

**Keywords:** digital twin, epistemology, falsifiability, causal inference, counterfactuals, dynamical systems, validation, uncertainty, regulatory science, precision medicine

## Abstract

**Objective:**

To clarify how validation requirements should be specified for medical digital twins used in clinical decision support, particularly when such systems are intended to compare interventions, treatment timings, dosages, or sequential care strategies.

**Perspective:**

Medical digital twins are heterogeneous systems that may combine prediction, simulation, mechanistic modeling, machine learning, data assimilation, uncertainty quantification, and decision-support functions. Their evaluation should therefore be driven by their intended use rather than by a single definition of what a digital twin is. For digital twins used primarily for visualization, monitoring, or short-term forecasting, predictive accuracy, calibration, discrimination, and robustness may be the central validation targets. However, when digital twins are used to support intervention-oriented clinical decisions, retrospective accuracy under historical clinical practice is insufficient on its own.

**Key message:**

Intervention-oriented digital twins address action-conditioned questions: what is predicted to happen under specified alternative actions, assumptions, time horizons, and clinical contexts. Their validation should therefore extend beyond scalar performance metrics to include uncertainty representation, updating stability, robustness under regime change, action-regime validity, counterfactual consistency, clinically weighted error, and decision-level consequences. This requires drawing on established traditions in forecast verification, causal inference, uncertainty quantification, model verification and validation, decision theory, control theory, and post-deployment monitoring. The level of causal or mechanistic support required should match the clinical claim being made, whether at the genotype, phenotype, physiological, or care-process level.

**Conclusion:**

The scientific-instrument framing is proposed as a pragmatic validation lens for intervention-oriented digital twins, not as a universal definition of digital twins. It helps define the scope within which their outputs can support clinical reasoning. Medical digital twins should be accompanied by explicit validation statements specifying their target population, prediction horizon, supported interventions, uncertainty bounds, and known failure conditions.

## Graphical abstract

**Graphical Abstract:**
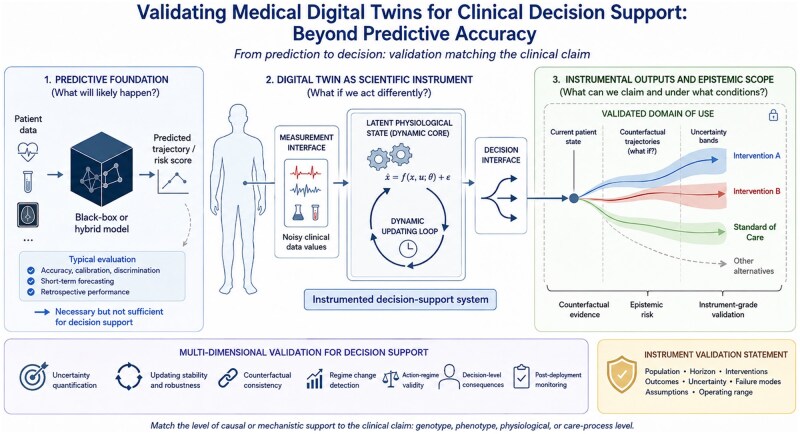

## Background and significance: validating intervention-oriented medical digital twins

Digital twins are increasingly proposed as tools for personalized medicine, with the aim of creating computational representations of patients that integrate data, update over time, and support prognosis, monitoring, simulation, or therapeutic planning.^1^^,^^2^ In medicine, however, the term “digital twin” covers heterogeneous systems, including descriptive replicas, synchronized simulators, mechanistic models, machine-learning forecasters, in silico trial platforms, and clinical decision-support tools.^3–6^ This heterogeneity is not a weakness: different clinical uses require different architectures and validation standards. The central issue is therefore not to impose a universal definition of medical digital twins, but to clarify what evidence is required for a given use case. Predictive performance remains necessary. A digital twin that cannot reproduce observed trajectories, remain calibrated, or anticipate clinically relevant outcomes is unlikely to be useful.^7^ However, predictive accuracy evaluated under historical clinical practice may be insufficient when the twin is used to compare interventions, treatment timings, dosages, or sequential care strategies. In such settings, the relevant question is not only what is likely to happen under current practice, but what is predicted to happen under specified alternative actions, assumptions, and time horizons.^8^ This distinction is well recognized across causal inference, uncertainty quantification, decision theory, model validation, dynamic treatment regimes, control theory, and forecast verification.^9–18^ The contribution of this article is therefore not to introduce a new validation theory or to claim that all digital twins must be causal models or scientific instruments. Rather, it synthesizes established validation principles for a specific subset of medical digital twins: systems dynamically linked to patients or populations, updated with data over time, and used to support intervention-oriented clinical decision-making.

We use the term “scientific instrument” pragmatically, as a validation lens rather than as a new definition of digital twins. When a digital twin informs clinical action, its outputs should be interpreted as conditional, model-mediated evidence whose reliability depends on assumptions, calibration, operating range, uncertainty representation, updating stability, and context of use.^19–22^ This framing does not imply that all medical digital twins require the same evidentiary burden. Twins used for visualization, education, descriptive monitoring, or short-term forecasting may be evaluated primarily through predictive performance, calibration, usability, and robustness. Twins used to recommend, compare, or optimize clinical actions require additional evidence because their outputs may influence treatment decisions and patient trajectories. Recent work has explicitly framed digital twins as part of the broader P4 medicine paradigm, emphasizing their role in predictive, preventive, personalized, and participatory care.^23^ In this view, digital twins are not a single method but a flexible paradigm combining patient-specific data, mechanistic modeling, dynamic updating, virtual intervention, explainability, and learning over time. This article is consistent with this broad framing but focuses on a narrower question: what validation evidence is required when such systems are used to support intervention-oriented clinical decisions?

The central principle is that the validation burden should match the decision claim. For intervention-oriented digital twins, evaluation should extend beyond scalar accuracy metrics to include verification, validation for the intended use, uncertainty quantification, robustness under regime change, stability of updating, and decision-level consequences. The relevant question is therefore not simply whether the model is accurate, but for what decision, under what assumptions, over what time horizon, and within what operating range the twin can be considered valid.

## Computational physiology, forecast verification, and medical digital twins

Long before the current terminology of “medical digital twins” became widespread, patient-specific physiological modeling had already been formulated through inverse problems, probabilistic inference, and quantitative differential diagnosis, notably in the work of Zenker, Rubin, and Clermont on mathematical physiology.^24^ Related traditions in critical illness modeling and translational systems biology, including work by An and colleagues on Critical Illness Digital Twins, have emphasized fit-for-purpose modeling, mechanistic simulation, uncertainty quantification, verification, validation, and control-oriented uses of digital twins in sepsis and acute illness.^25–28^ In parallel, data assimilation in medicine has shown how mechanistic models, Bayesian inference, sparse clinical observations, and sequential updating can be combined to forecast future states, infer unobserved physiological variables, and phenotype patients, particularly in glucose–insulin and ICU contexts.^25^^,^^26^^,^^29^ The evaluation of such systems also builds on mature traditions in forecast verification and device assessment, where predictions are judged not only by numerical error but by calibration, reliability, uncertainty, sharpness, clinical utility, and decision consequences.^29^ This broader context is also reflected in the recent JAMIA focus issue on interdisciplinary computational methods for clinical applications, which highlights the need to connect biomedical informatics, mathematical modeling, machine learning, data assimilation, causal reasoning, and clinical deployment.^25^^,^^30–32^ The present article therefore does not propose a new definition of digital twins, nor does it claim novelty for decision-oriented validation. Rather, it uses the “scientific instrument” framing as a pragmatic validation lens for a specific class of medical digital twins: systems dynamically linked to patients or populations, updated with data over time, and used to support intervention-oriented clinical decision-making.

## Scope and architecture of intervention-oriented medical digital twins

### Beyond prediction and simulation: digital twins as layered decision-support systems

This section does not seek to redefine digital twins or to exclude predictors, simulators, black-box models, or foundation-model-based systems from the category. Under broad definitions, a medical digital twin may include predictive models, mechanistic simulators, statistical models, machine-learning systems, control-oriented architectures, or hybrid combinations of these approaches.^4–6^ This broad position is consistent with engineering and mathematical frameworks such as Asch’s *A Toolbox for Digital Twins*,^33^ which presents digital twins as systems coupling computational models, data assimilation, inverse problems, uncertainty quantification, optimization, and machine learning to support prediction and decision-making. Accordingly, prediction, simulation, and data-driven components should not be viewed as exclusions from the category of digital twins. Our narrower focus is instead on the additional validation evidence required when these components are assembled into systems intended to support intervention-oriented clinical decisions.

Prediction and simulation are often essential components of digital twins and may be sufficient for monitoring, forecasting, visualization, or exploratory modeling.

The relevant issue is not whether a system is “really” a digital twin, but what validation evidence is required for the claim made from its outputs. A twin used for monitoring or short-term forecasting may be evaluated through predictive accuracy, calibration, discrimination, trajectory error, usability, and robustness to missingness. By contrast, when a twin is used to compare interventions, explore treatment timing, optimize dosage, or support sequential clinical decisions, prediction and simulation become part of a broader decision-support architecture. In this setting, validation must also address uncertainty, updating stability, robustness under regime change, and the consequences of the induced decision policy.

For intervention-oriented clinical decision support, a useful digital twin architecture typically combines 4 elements: a representation of the relevant physiological or clinical state, a measurement or synchronization process linking the model to observed data, an updating mechanism that revises the twin over time, and an objective or decision layer defining the clinical action being evaluated.^19^^,^^20^ This does not constitute a universal definition of digital twins; rather, it specifies the components that become important when the twin is used to support action-oriented clinical reasoning.

Intervention-oriented questions are still predictive questions, but they are predictions indexed to specified actions, time horizons, outcomes, and assumptions. The validation challenge is therefore not that prediction is irrelevant, but that predictions under alternative actions require evidence beyond accuracy under historical practice. The scientific issue is evidential rather than taxonomic: the validation burden should be determined by the intended use of the digital twin and by the strength of the clinical claim made from its outputs.

### A possible formal architecture for intervention-oriented twins

Let a patient’s latent physiological state at time t be $s_{t}\in S$, observations be $o_{t}\in O$, and clinical actions (treatments, interventions) be $a_{t}\in A.$^34^^,^^35^ For the intervention-oriented use case considered here, one possible formal architecture can be expressed as the tuple:


$$T=\left(f_{\theta },\;g_{\phi },\;\Pi ,\;U\right),$$

where:

-
$f_{\theta }$ is a state transition model (dynamics),
-
$g_{\phi }$ is a measurement model linking latent states to observations,
-
Π is an updating rule (data assimilation/inference),
-
U is a utility or objective functional defining what “optimal” means clinically.

A common instantiation is:


$$s_{t+1}=f_{\theta }(s_{t},a_{t},\varepsilon_{t}),o_{t}=g_{\phi }(s_{t},\eta_{t})$$

with stochastic terms $\varepsilon_{t},\eta_{t}$capturing process and measurement noise.

The twin’s personalization is not merely parameter fitting^36^; it is a continual inference problem:


$$p(s_{0:T},\theta ,\phi |o_{0:T},a_{0:T})\propto p(\theta ,\phi )\prod_{t=0}^{T-1}p(s_{t+1}|s_{t},a_{t},\theta )\prod_{t=0}^{T}p(o_{t}|s_{t},\phi )$$

and an updating rule Π implementing filtering/smoothing (Bayesian, variational, ensemble, or particle).^37^

This definition makes the digital twin an instrumented system: it has dynamics, measurements, updating, and objectives, like an experimental apparatus coupled to readouts and calibration procedures.

This tuple should not be read as a universal definition of all digital twins. Rather, it specifies the components that become relevant when a medical digital twin is used to compare actions, simulate alternative clinical trajectories, or support sequential decision-making.

### Dynamic updating and instrument drift

For medical digital twins that are updated over time, the updating rule Πdetermines how new observations revise the inferred patient state and model parameters. Formally, this can be written as:


$$p(s_{t},\theta |o_{0:t})=\Pi (p(s_{t-1},\theta |o_{0:t-1}),o_{t}),$$

where $s_{t}\;$denotes the latent physiological or clinical state at time t, θ denotes the model parameters, $o_{0:t}$ denotes the observations accumulated up to time t, and Π denotes the updating or data-assimilation rule. This rule determines whether the twin remains calibrated, adapts to clinical change, or progressively diverges from the patient or population it represents.^35^^,^^38^^,^^39^

This temporal dependence creates a specific validation challenge: drift. Small biases in the updating process, arising from mis-specified measurement noise, delayed observations, inappropriate priors, missingness, or unmodeled clinical change, can accumulate over time.^36^^,^^40^ The twin may then remain internally coherent while its inferred state becomes increasingly misaligned with the underlying clinical system. This is particularly relevant because both biology and care processes are non-stationary: disease progression, treatment effects, behavioral adaptation, and measurement practices may all change over time.^12^^,^^41^ Drift can occur when the assumed observation or transition model no longer matches the true data-generating process. In such cases, posterior updates may become biased,


$$E_{\Pi }[s_{t}]\neq E_{p^{*}}[s_{t}],$$

where $E_{\Pi }[s_{t}]$ denotes the state expected under the twin’s updating rule and $E_{p^{*}}[s_{t}]$ denotes the expected state under the true, usually unobserved, data-generating process.^42^^,^^43^

**Figure 1.**
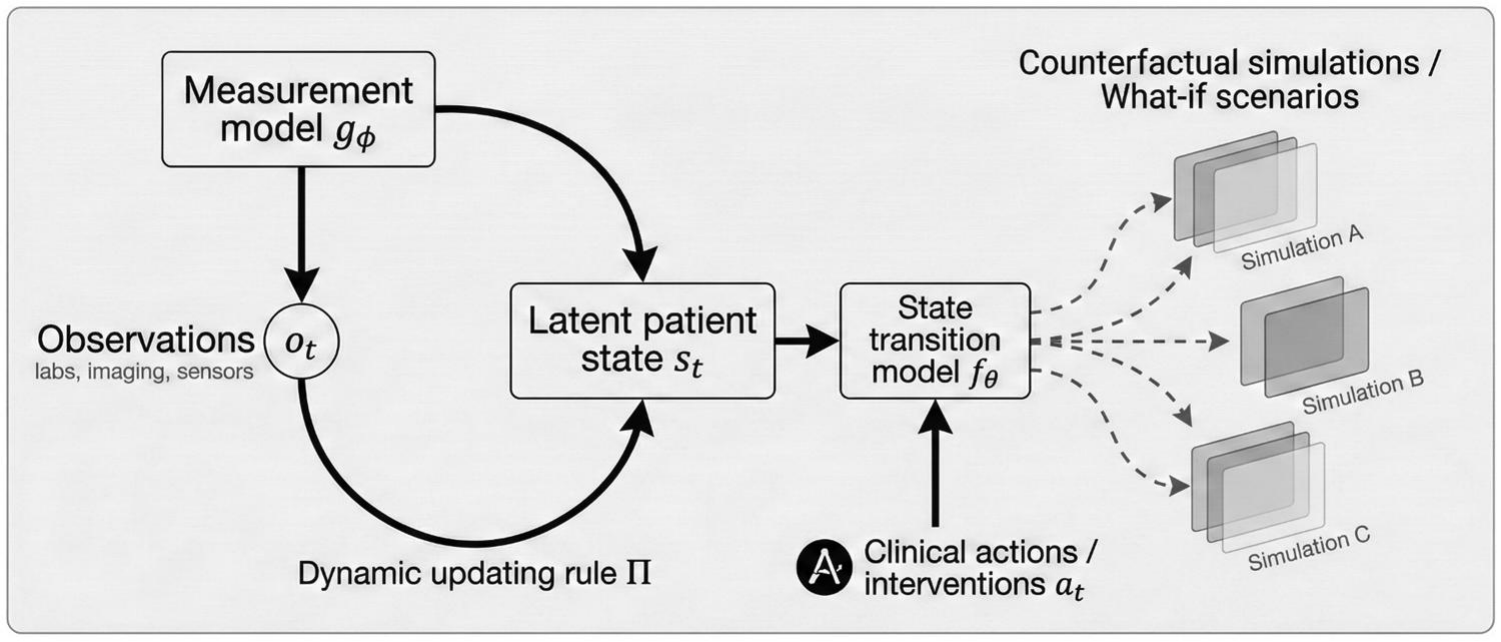
The digital twin as a scientific instrument: structure, updating, and drift. Conceptual representation of a medical digital twin conceived as a scientific instrument rather than a predictive model. The twin is composed of 4 coupled elements: (1) a latent state transition model representing individual physiological dynamics; (2) a measurement model linking latent states to noisy and incomplete clinical observations; (3) a dynamic updating rule that assimilates new data over time; and (4) a decision objective defining clinically relevant actions. Continuous updating maintains the coupling between the twin and the patient, enabling individualized counterfactual simulations. However, mis-specification of the measurement or updating processes can induce *instrument drift*, whereby the twin remains internally coherent and confident while progressively diverging from the patient’s true physiological state. This drift illustrates a central source of epistemic risk that is not detectable through static predictive accuracy alone.

This problem is analogous to calibration drift in measurement systems, but in digital twins it affects inferred states, uncertainty estimates, and potentially action-conditioned predictions.^20^^,^^44^ Conventional predictive metrics may not detect this failure mode. A twin can retain acceptable short-term accuracy under historical treatment policies while becoming unreliable under changes in measurement frequency, clinical practice, or treatment regime.^45^^,^^46^ Detecting drift therefore requires explicit evaluation of the updating process itself, including sensitivity to observation frequency, robustness to missingness, calibration over time, and stability under regime change (Figure 1).^45^^,^^47^ Predictive models, simulators, and digital twins often overlap in practice, and may share evaluation metrics (Table 1).^14^^,^^15^

**Table 1.** Conceptual and epistemic comparison of predictive models, simulators, and digital twins as scientific instruments.

Dimension	Predictive model	Simulator	Digital twin (scientific instrument)
Primary object	Statistical estimator	Generative dynamical system	Patient-indexed experimental system
Ontological status	Mapping from data to outcome	Abstract system representation	Instrument mediating theory and patient
Reference entity	Population-level patterns	Generic or archetypal system	Specific individual (indexed to reality)
Temporal structure	Often static or shallow dynamics	Explicit dynamics	Explicit, adaptive, individualized dynamics
Personalization	Parameter conditioning	Optional parameter tuning	Continuous data assimilation
Update mechanism	Retraining	Reparameterization	Bayesian/sequential updating
Intervention support	Implicit or absent	Explicit but generic	Explicit, individualized, sequential
Counterfactual reasoning	Not well-defined	Possible but unanchored	Central, patient-specific
Primary epistemic goal	Prediction	Reproduction of behavior	Knowledge production under intervention
Typical question answered	“What will likely happen?”	“How does this system behave?”	“What is the predicted trajectory or outcome distribution for this patient under action A versus action B, given stated assumptions?”
Core assumption	Stationarity, exchangeability	Structural completeness	Causal sufficiency + observability
Main source of error	Statistical bias/overfitting	Model misspecification	Epistemic risk, causal error
Validation target	Predictive accuracy	Fidelity to known dynamics	Counterfactual credibility
Validation metrics	AUC, RMSE, calibration	Trajectory similarity, stability	Invariance, calibration, policy safety
Falsifiability mode	Poor out-of-sample performance	Failure to reproduce dynamics	Violation of causal/temporal constraints
Clinical role	Risk stratification	Hypothesis exploration	Decision exploration under uncertainty
Regulatory framing	Algorithm	Scientific model	Medical decision instrument

Predictive models, simulators, and digital twins often overlap in practice and may share architectures, components, and evaluation metrics (Table 1).^6^^,^^15^^,^^16^ Accordingly, Table 1 should be read as a conceptual heuristic rather than a rigid taxonomy: a deployed medical digital twin may embed predictive models and simulators, and the same system may move along this spectrum depending on whether it is used for monitoring, hypothesis exploration, or action-oriented clinical decision support.

Their validation requirements depend less on the label assigned to the system than on the clinical claim made on the basis of its outputs. Predictive models are commonly evaluated by their ability to anticipate observed outcomes under historical data-generating processes,^9^ whereas simulators are assessed by their fidelity to known system behaviors and qualitative dynamics.^11^^,^^16^

For intervention-oriented digital twins, predictive performance remains necessary but should be complemented by evaluation of updating stability, uncertainty calibration, robustness under regime change, action-regime validity, and decision impact.^10^^,^^12^

## Digital twins as instruments: what instruments do that models do not

### Instruments as mediators between theory and reality

Scientific instruments occupy a distinctive epistemic position.^21^ They do not merely compute outputs from inputs, nor do they assert truths about the world in isolation. Rather, they mediate between theoretical constructs and empirical reality by rendering certain aspects of the world observable under controlled assumptions. A microscope does not predict the existence of cells; it operationalizes a theory of optics, imposes a measurement process, and produces a stream of evidence whose meaning depends on calibration, resolution, noise, and interpretation. The knowledge produced by an instrument is therefore conditional, structured, and intrinsically uncertain.^22^

Digital twins, when properly conceived, share this instrumental status. They do not simply output predictions about future outcomes. Instead, they instantiate a theoretical representation of an individual biological system, couple it to a measurement process, and generate evidence—simulated trajectories, posterior states, and counterfactual responses—that must be interpreted in light of explicit assumptions.^5^^,^^48^ Treating a digital twin as a model collapses this epistemic role into a narrow question of predictive accuracy. Treating it as an instrument foregrounds how knowledge is produced, constrained, and validated.

This distinction is not semantic. It has direct consequences for how digital twins should be designed, evaluated, and used in medicine.

### Extending the experimental space beyond physical feasibility

A defining feature of scientific instruments is their capacity to extend the experimental space.^3^^,^^49^ Telescopes enable observation beyond human vision; particle detectors reveal interactions inaccessible to direct manipulation. Digital twins similarly expand the experimental capacity of clinical science by enabling in silico interventions that cannot be performed physically on patients.^50–52^

Formally, a clinical intervention corresponds to an action $a_{t}$ applied to a system evolving according to latent dynamics,


$$s_{t+1}=f_{\theta }(s_{t},a_{t})+\varepsilon_{t}.$$

In real patients, only a single trajectory is observed, under a single sequence of actions constrained by ethics, feasibility, and irreversibility. A digital twin, by contrast, enables the exploration of alternative action sequences $\left\{a_{t}^{\left(k\right)}\right\}$ applied to the same inferred latent state $s_{t}$, producing counterfactual trajectories^53^


$$\{s_{t+h}^{\left(k\right)}{\}}_{h\geq 0}∼p_{\theta }(\cdot |s_{t},do(a_{t}^{\left(k\right)})).$$

This extension of the experimental space is not a metaphorical convenience; it is the central epistemic contribution of the digital twin. It allows clinicians and researchers to reason about timing, dosage, sequencing, and strategy in ways that are impossible to test directly at the individual level. However, because these experiments are simulated rather than physical, their evidential status depends entirely on the validity and scope of the underlying assumptions. The twin does not reveal what *will* happen, but what *would* happen if the modeled structure were correct.^54–56^

### Repeated trials and controlled exploration of decision sequences

Scientific instruments also enable repetition. Repeated measurements under controlled conditions are a cornerstone of experimental science, allowing variability to be characterized and hypotheses to be stress-tested.^57^ In medicine, repeated experimentation on the same individual is typically impossible.^58^ Digital twins partially overcome this limitation by supporting repeated in silico trials under fixed assumptions.^51^

Given a current posterior over latent states $p(s_{t}|D_{\leq t})$, a digital twin can simulate multiple realizations of future trajectories under a given policy π,


$$a_{t}=\pi (s_{t}),s_{t+1}∼f_{\theta }(s_{t},a_{t}),$$

thereby generating a distribution over outcomes rather than a single forecast. This capability transforms clinical reasoning from point prediction to distributional exploration.^59^ It allows systematic comparison of strategies, sensitivity analyses with respect to uncertainty, and evaluation of robustness to perturbations.^60^

Crucially, this form of repetition does not eliminate uncertainty; it makes it explicit. The resulting distributions are instrument readings, not empirical frequencies. Their interpretation requires calibration, validation, and discipline in uncertainty reporting. Without such discipline, repeated simulation risks producing an illusion of experimental control where none exists.

### Counterfactual evidence as an instrumental output

Perhaps the most distinctive epistemic product of digital twins is counterfactual evidence.^61^ Clinical decision-making is inherently counterfactual: choosing an action requires reasoning about outcomes that will not be observed.^8^ Digital twins make such reasoning explicit by generating estimates of interventional quantities,


$$E[Y|do(A=a),S_{t}=s],$$

rather than merely conditional associations $E[Y|A=a,S_{t}=s]$.

This capability marks a fundamental departure from conventional predictive models.^52^^,^^59^^,^^62^ A predictor estimates what is likely to occur under the observed policy; an instrumented digital twin estimates what would occur under alternative policies. The resulting outputs—simulated responses, dose-response curves, and timing effects—are not ground truth. They are conditional evidence produced by an instrument operating under explicit causal and dynamical assumptions.^48^^,^^63^

Recognizing this status is essential. Counterfactual outputs must be calibrated, bounded by uncertainty, and interpreted within a declared domain of validity.^64^^,^^65^ Treating them as factual predictions collapses the distinction between evidence and reality, inviting overconfidence and misuse.

### Epistemic consequences of the instrumental framing

The instrumental framing of digital twins carries direct epistemic consequences.^61^

- First, it reclassifies the twin’s outputs as measurements rather than truths. Like any instrument, a digital twin can be miscalibrated, biased, or operate outside its valid range.
- Second, it implies that validation must target properties such as calibration, invariance, and robustness under intervention, rather than accuracy alone.
- Third, it demands interpretive discipline: the twin’s outputs acquire meaning only in conjunction with clinical expertise and contextual knowledge.

In this sense, digital twins are neither oracles nor autonomous agents. They are epistemic devices that expand what can be asked, explored, and tested in medicine.^1^^,^^5^^,^^62^ Their power lies not in replacing human judgment, but in restructuring the evidential landscape within which judgment is exercised. Treating digital twins as instruments rather than models is therefore not a rhetorical choice; it is a necessary condition for their scientific and clinical legitimacy.

## What knowledge digital twins produce: 3 epistemic products

Digital twins do not merely improve prediction; they produce distinct forms of scientific knowledge that are poorly captured by conventional modeling frameworks.^3^^,^^49^ When conceived as instruments, digital twins generate epistemic products that are counterfactual, temporal-mechanistic, and conditional. These products differ both in nature and scope from the outputs of predictive models, and they require distinct criteria of interpretation and validation.

### Counterfactual knowledge

Many treatment decisions can be expressed as action-conditioned prediction problems. For example, a question such as “what would happen if treatment A rather than B were administered?” becomes operational when one specifies the target outcome, time horizon, action regime, current patient state, and assumptions linking actions to outcomes. In this sense, intervention-oriented reasoning is not opposed to prediction; it requires predictions whose validity depends on the action regime under which they are made.^8^^,^^66^^,^^67^ The challenge is that predictions learned and validated only under historical clinical practice may not remain valid when used to compare alternative interventions, timings, or policies. In formal causal terms, this reasoning concerns contrasts between potential outcomes,


$$E[Y^{a}-Y^{a^{\prime }}|X=x],$$

where $Y^{a}$ denotes the outcome that would be realized under intervention a, and X represents baseline patient characteristics. Using Pearl’s do-notation, the same quantity can be expressed as


E[Y∣do(A=a),X=x],

which explicitly distinguishes intervention from observation.

Predictive models, even when highly accurate, typically estimate conditional expectations of the form


E[Y∣A=a,X=x],

which coincide with the interventional quantity only under strong and often unverifiable assumptions, such as the absence of unmeasured confounding. In routine clinical data, where treatment assignment is shaped by disease severity, clinician judgment, and institutional constraints, this equivalence rarely holds.^68^ Prediction is not inherently correlational. Mechanistic, causal, and control-oriented models may generate predictions that are explicitly action-conditioned. The limitation arises when predictions are learned and validated only under historical clinical practice, without specifying whether they remain valid under alternative interventions or policies.

A digital twin conceived as an instrument is explicitly designed to approximate the interventional query rather than the observational one.^69^ By embedding causal assumptions into its structure and updating its state based on patient-specific data, the twin produces estimates of unobserved outcomes under alternative actions. This counterfactual knowledge is not guaranteed to be correct, but it is epistemically distinct: it addresses a question that prediction alone cannot answer. The scientific value of the digital twin therefore lies not in asserting counterfactual truths, but in making counterfactual reasoning explicit, structured, and open to falsification.^53^

### Temporal-mechanistic knowledge

Biological systems are dynamic, history-dependent, and often governed by delayed responses. In many clinical contexts, risk is not determined solely by the magnitude of a physiological variable, but by its temporal pattern: the speed of change, the duration of exposure, or the phase relationship between interacting processes.^70^^,^^71^ Conventional models frequently treat such temporal features as nuisances to be smoothed or averaged away. Digital twins, by contrast, can encode time explicitly as a causal dimension.

This capacity is formalized by dynamical representations that incorporate memory, delay, and stochasticity, such as


$$ds_{t}=b_{\theta }(s_{t},s_{t-\tau },a_{t})\;dt+\Sigma_{\theta }(s_{t})\;dW_{t},$$

where the current state $s_{t}$ depends not only on present inputs but also on past states $s_{t-\tau }$, and where stochastic fluctuations reflect intrinsic biological variability rather than measurement noise alone. Such formulations allow the digital twin to represent phenomena such as delayed toxicity, cumulative exposure effects, or destabilization of regulatory loops.

The knowledge produced by these structures is temporal-mechanistic. It concerns not only whether an event is likely to occur, but also when, under what timing of interventions, and with what stability properties. Phase shifts, lag distributions, and recovery kinetics become objects of inference rather than residual artifacts. In this sense, digital twins enable knowledge claims about timing itself as a causal driver of clinical outcomes, extending beyond the static risk estimates produced by conventional models.^72^

### Conditional mechanistic knowledge: “if–then” laws

Digital twins rarely discover universal biological laws in the classical sense.^19^ Their contribution is more often conditional: they specify mechanistic or causal assumptions and explore what follows if those assumptions hold.^73^ The resulting outputs should therefore be interpreted as “if–then” claims: if the modeled structure is adequate for the intended context, then these trajectories, responses, or failure modes are expected.

This conditional mode of reasoning is well established in theoretical physics, systems biology, and control theory, where models are used to derive implications whose validity is delimited empirically rather than assumed universally.^74^ In medicine, digital twins extend this logic to individualized systems where direct experimentation is constrained and mechanisms are only partially observed.^72^

The credibility of such claims depends on whether assumptions are explicit and testable through observed trajectories, regime changes, calibration checks, or targeted falsification strategies.^9^ Conditional mechanistic outputs are therefore useful only within a declared scope of validity. They should be interpreted as model-mediated evidence under stated assumptions, not as direct statements of biological truth.

## Falsifiability: when can a digital twin be wrong?

For intervention-oriented digital twins, falsifiability should not be reduced to poor prediction on held-out data. Because such systems may be used to simulate interventions and counterfactual trajectories, they should make claims that generate testable implications in observable data. A digital twin is scientifically useful only if its assumptions imply patterns that could fail, such as calibration across subgroups and time horizons, temporal signatures, interventional response shapes, or invariance constraints under changes in clinical context or treatment regime.

A minimal falsification strategy therefore combines calibration and invariance testing. Calibration asks whether predicted distributions agree with observed outcomes across relevant subgroups, time windows, and settings. Invariance testing asks whether the mechanisms encoded by the twin remain stable when measurement schedules, treatment regimes, or clinical contexts change.^10^^,^^75^ Violations of these constraints do not necessarily invalidate the entire model, but instead delimit its operating domain and identify where counterfactual outputs should not be trusted.

Counterfactual claims cannot be directly observed for the same patient under mutually exclusive interventions.^76^ However, their implications can be assessed indirectly using natural experiments, quasi-random variation, regime changes, off-policy evaluation, or prospective studies in which selected twin-informed policies are tested.^72^ The goal is not to prove that the twin is universally correct, but to make its assumptions explicit, expose them to possible failure, and define the domain within which its outputs can support clinical reasoning. The required level of causal or mechanistic support should match the decision claim being made. Genotype-level causality is necessary when the twin supports molecularly targeted interventions or genomic treatment selection, whereas phenotype-level, physiological, or care-process mechanisms may be sufficient for decisions about monitoring, risk stratification, dosing, timing, or sequential treatment policies. Thus, an intervention-oriented digital twin does not need to be causally complete at every biological scale, but it should make explicit which level of organization supports each clinical claim.^10^^,^^17^^,^^18^^,^^24^^,^^29^^,^^77^^,^^78^

## Epistemic risk: the central failure mode of in silico medicine

### From statistical risk to epistemic risk

The evaluation of machine-learning systems in medicine is often framed in terms of statistical risk.^79^ For a predictor $\hat{f}$ mapping inputs X to outcomes Y, performance can be expressed as the expected loss under the empirical data-generating distribution:


$$R(\hat{f})=E[\ell (Y,\hat{f}(X))].$$

This formulation is appropriate when the main objective is prediction and when future data are expected to resemble the conditions observed during training.^80^ For intervention-oriented digital twins, however, predictive error must be complemented by evaluation of the assumptions that support action-conditioned predictions.^72^ These systems are often used to compare interventions, simulate alternative trajectories, or support sequential decisions in which actions influence future states, observations, and outcomes.^17^ We refer to epistemic risk as the risk that a digital twin produces plausible, internally coherent, but misleading outputs because its structural assumptions, causal relationships, measurement model, or updating process are incorrect.^81^

This risk arises when uncertainty about mechanisms, observability, or causal structure is treated as random error alone.^82^ A twin may therefore appear calibrated under historical practice while being unreliable under alternative treatment regimes or decision policies.^83^ One way to summarize this problem is to view epistemic risk as a combination of decision error and confident wrongness:


$$R_{E}(T)=E[\Delta (\pi_{T},\pi^{*})]+\lambda E[\mathrm{Conf}(T)\cdot \mathrm{Wrong}(T)].$$

Here, $\pi_{T}$ denotes the policy induced by the digital twin, while $\pi^{*}$ represents a reference policy under correct interventional reasoning. The first term captures divergence between the twin-induced and reference policies. The second term penalizes confident but wrong recommendations, a clinically important failure mode because uncertainty can be incorporated into judgment, whereas precise but misleading outputs may directly shape unsafe decisions.^84^ Importantly, minimizing statistical risk does not necessarily reduce epistemic risk; in some cases, improvements in predictive accuracy may even increase confident wrongness by masking structural misspecification.

This formulation is not intended as a universal metric, but as a conceptual reminder that minimizing predictive loss does not necessarily minimize decision-relevant error. For digital twins used to inform clinical action, validation should therefore assess not only predictive performance, but also calibration, robustness, uncertainty representation, falsifiability, auditability, and decision-level consequences.^85^^,^^86^

### Clarifying action versus observation: selection bias and the risk of reinforcing clinical practice

A central distinction in causal inference is the difference between observing a treatment and acting on it.^17^ Formally, this separates conditional associations,


E[Y∣A=a,X=x],

from interventional quantities,


E[Y∣do(A=a),X=x],

where Y denotes the outcome, A denotes the treatment or clinical action, a denotes a specific treatment value, X denotes baseline covariates, and do(A=a) denotes the hypothetical intervention that actively sets treatment A to a, rather than merely observing that treatment assignment.^77^

This distinction is critical in clinical data because treatments are rarely assigned at random.^87^ They are often given according to disease severity, prognosis, clinician judgment, or institutional practice.^88^ If S denotes an unobserved or partially observed severity state influencing both treatment assignment and outcome, the structure can be summarized as:


A←S→Y.

In this case, S acts as a confounder: treatment intensity may become a proxy for baseline severity rather than a clean estimate of treatment effect.^89^^,^^90^ A digital twin trained primarily on observational data may therefore learn the prognosis of patients who historically received a treatment, rather than the effect of assigning that treatment.^91^ In this regime, the twin does not estimate the effect of treatment; it estimates the prognosis of patients who historically received that treatment.

The epistemic risk arises when this observational association is interpreted as an intervention-oriented claim.^92^^,^^93^ For example, a twin may infer that aggressive treatment worsens outcomes not because the treatment is harmful, but because it was historically given to the sickest patients.^94^ Strong predictive performance under historical practice does not resolve this problem: a twin may remain well calibrated for observed care while being unreliable under alternative treatment regimes.^83^^,^^95^ In such cases, apparent accuracy can increase confidence in biased counterfactual outputs.^81^^,^^96^

For intervention-oriented use, digital twins should therefore distinguish actions from observations at the structural level. Treatments should be modeled as clinical actions, severity should be represented as part of the latent state when possible, and validation should test behavior under changes in treatment regime rather than only under historical associations.^97^

### Model-induced illusion: when plausibility masks causal error

A further source of epistemic risk is what we call model-induced illusion: the situation in which a digital twin produces trajectories that are physiologically plausible, internally coherent, and apparently well calibrated, yet remain misleading for intervention-oriented reasoning.^83^^,^^92^ This failure mode reflects a mismatch between observational adequacy and interventional validity.^10^^,^^17^ Consider a digital twin estimating outcomes through the structural relation,


$$Y_{t}=h_{\theta }(S_{t},A_{t})+\epsilon_{t},$$

where $Y_{t}$ denotes the outcome at time t, $S_{t}$ denotes the latent physiological state, $A_{t}$ denotes the clinical action, $h_{\theta }$ denotes the modeled response function with parameters θ, and $\epsilon_{t}$ denotes the residual error. Good prediction under the observed treatment policy,


$${\hat{Y}}_{t}\approx Y_{t}\text{for}\;A_{t}∼p_{\mathrm{obs}}(A_{t}|S_{t}),$$

does not guarantee validity under an alternative intervention $do(A_{t}=a)$. The model may reproduce historical trajectories while misestimating what would happen if treatment policy changed.

Several mechanisms can produce this illusion. Unmeasured confounding may distort the learned response function: if unobserved factors $U_{t}$influence both treatment assignment and outcome, the twin may approximate


$$h_{\theta }(S_{t},A_{t})\approx E[Y_{t}|S_{t},A_{t}],$$

rather than the desired interventional quantity $E[Y_{t}|do(A_{t}),S_{t}].$^87^ In this case, counterfactual outputs may inherit the biases of historical clinical practice.

Mis-specified measurement models can bias the inferred latent state. If observations are modeled as


$$o_{t}=g_{\phi }(S_{t})+\eta_{t},$$

where $o_{t}$ denotes observed clinical data, $g_{\phi }$ denotes the measurement model, and $\eta_{t}$ denotes measurement error, then sensor drift, selective measurement, or care-driven observation schedules may cause the inferred state ${\hat{S}}_{t}$ to deviate from the patient’s true physiology.^98^ Apparent calibration may then reflect consistency with the measurement process rather than validity of the underlying physiological representation.^81^

Temporal mis-specification can preserve short-term accuracy while undermining intervention validity.^99^ Biological systems often involve delay, memory, and path dependence, which may be represented as:


$$S_{t+1}=f_{\theta }(S_{t},S_{t-\tau },A_{t})+\varepsilon_{t},$$

where $S_{t-\tau }$ represents a delayed state and $\varepsilon_{t}$ denotes process noise. If such temporal structure is omitted or incorrectly parameterized, the twin may fit observed trajectories while failing under interventions that alter timing or sequencing.^100^ Finally, digital twins may optimize surrogate utilities,


$$U=E[u(Y_{t})],$$

where $u(Y_{t})$ represents the utility assigned to the predicted outcome. If this utility reflects short-term or easily measurable endpoints rather than the true clinical objective, the twin may recommend actions that improve intermediate markers while worsening longer-term outcomes.^95^^,^^97^ Model-induced illusion is therefore not simply poor prediction. It is a failure in which plausible simulations conceal biased causal structure, mis-specified measurement, inadequate temporal dynamics, or misaligned objectives. Detecting it requires validation strategies that test causal structure, temporal robustness, calibration under regime change, and decision consequences, rather than relying on predictive accuracy alone.^82^

## Predictive accuracy as one layer of digital twin validation

### Forecast evaluation as the starting point for digital twin validation

Predictive performance is indispensable for digital twin validation, but mature evaluation traditions have long shown that prediction should not be judged by scalar accuracy alone. Forecast verification evaluates predictions through accuracy, reliability, calibration, resolution, sharpness, uncertainty, and decision value.^101^^,^^102^ In probabilistic forecasting, predictive distributions should be as sharp as possible while remaining calibrated, a principle directly relevant to digital twins that output trajectories, posterior states, or risk distributions.^103^ Similarly, glucose error grids and continuous clinical value prediction show that errors of similar numerical magnitude may have different clinical consequences depending on context, timing, and actionability.^79^^,^^104^ Uncertainty quantification is also central, because uncertainty in a digital twin may arise from parameters, measurement noise, model structure, numerical approximation, missing data, and incomplete observability.^105^ Probabilistic numerics further treats numerical approximation itself as a source of uncertainty, which is relevant for twins relying on differential equations, stochastic simulation, Bayesian filtering, or data assimilation.^106^ Model verification and validation provide an additional distinction: verification asks whether the computational model has been implemented correctly, whereas validation asks whether it is adequate for a specified purpose, context, and domain of use.^107^ Conventional metrics such as RMSE assess agreement between predicted and observed outcomes:


$$\mathrm{RMSE}=\sqrt{E[(Y-\hat{Y}{)}^{2}]},$$

where Y denotes the observed outcome and $\hat{Y}$ denotes the predicted outcome. Discrimination metrics evaluate whether the model separates outcome classes conditional on observed covariates.^108^ These metrics remain necessary for assessing predictive adequacy, but they may be insufficient when a digital twin is used to compare interventions or support sequential decisions.^72^

The limitation is that accuracy is usually evaluated under the observational distribution p(Y∣X,A), where X denotes covariates and A denotes observed clinical actions.^10^ When the twin is used to reason about alternative actions, the relevant quantity may instead be interventional:


E[Y∣do(A=a)]≠E[Y∣A=a],

where do(A=a) denotes an intervention that actively sets action A to value a. This distinction matters in the presence of confounding, feedback, temporal dependencies, or policy changes.^87^^,^^97^

**Figure 2.**
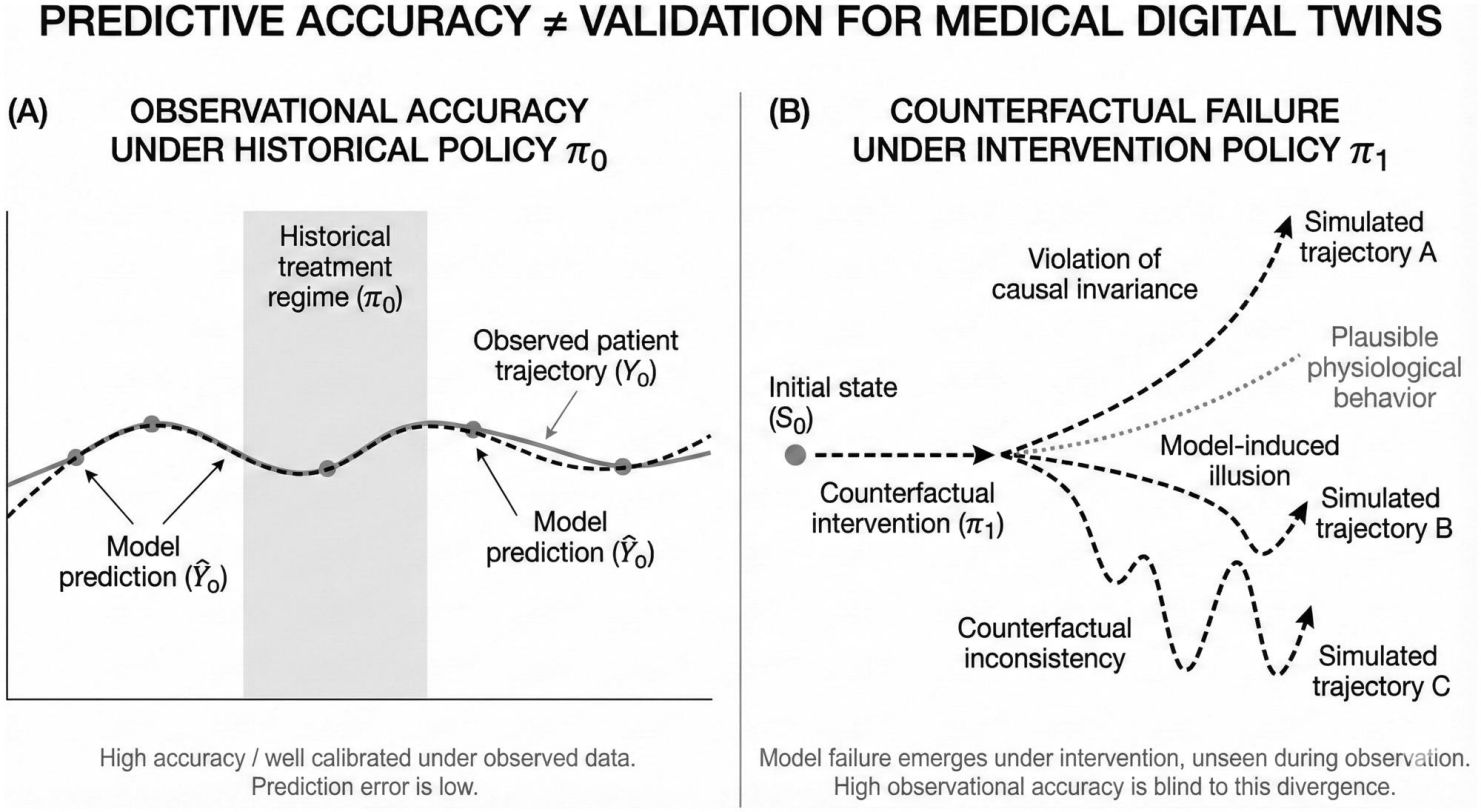
Why predictive accuracy is insufficient: epistemic risk and instrument-grade validation. Schematic comparison between accuracy-based validation (A) and instrument-grade validation for medical digital twins (B). Conventional evaluation focuses on predictive performance under the historical data-generating process (eg, AUC, RMSE), which may remain high even when causal structure, temporal dynamics, or uncertainty calibration are incorrect. In contrast, instrument-grade validation interrogates multiple epistemic dimensions: measurement validity, dynamical adequacy, calibration of uncertainty, counterfactual consistency under intervention, and safety of induced decision policies. The figure illustrates how a digital twin can appear accurate yet generate misleading counterfactuals when deployed under policy or regime change, highlighting epistemic risk as the dominant failure mode in interventional use.

Therefore, the concern is not prediction itself, but the use of retrospective predictive accuracy as sufficient evidence for intervention-oriented digital twins. When a twin is used to compare actions, treatment timings, or sequential policies, predictive evaluation should be complemented by uncertainty assessment, updating stability, robustness under regime change, clinically weighted error analysis, and evaluation of decision-level consequences^81^^,^^83^ (Table 2 and Figure 2).

**Table 2.** Established evaluation traditions informing the validation of intervention-oriented medical digital twins.

Established tradition	Core contribution	Relevance for medical digital twins
Model verification	Checks whether the computational model is implemented correctly	Ensures that the twin’s code, numerical solvers, updating routines, and data pipelines behave as intended
Model validation	Assesses whether the model is adequate for a specified purpose and domain of use	Clarifies what the twin is valid for, in which population, over which horizon, and under which assumptions
Calibration	Evaluates agreement between predicted probabilities or distributions and observed outcomes	Ensures that the twin’s uncertainty estimates are interpretable and not systematically overconfident
Forecast verification	Evaluates accuracy, reliability, sharpness, resolution, uncertainty, and decision value	Provides a mature vocabulary for assessing digital twin predictions beyond scalar error
Uncertainty quantification	Characterizes uncertainty from parameters, structure, measurements, numerical approximation, and data limitations	Helps distinguish uncertainty that can be propagated from uncertainty that remains structurally unmodeled
Probabilistic numerics	Treats numerical approximation and computation itself as a source of uncertainty	Relevant when differential equations, stochastic simulations, or data assimilation are used within the twin
Decision theory	Evaluates predictions according to expected utility, loss, preferences, and consequences of action	Links twin outputs to the clinical decisions they are intended to support
Control theory	Evaluates sequential actions, feedback, stability, safety constraints, and policy performance	Essential for twins used to guide adaptive treatment, closed-loop care, or repeated decision-making
Clinically weighted error metrics	Weights prediction errors by their potential clinical consequences	Prevents numerically small but clinically dangerous errors from being treated as benign
Post-deployment monitoring	Tracks drift, recalibration needs, and performance changes after deployment	Supports continuous surveillance of digital twins as learning or updating systems

### Instrument-grade validation as multi-dimensional interrogation

For intervention-oriented digital twins, validation should assess the full decision-support system, not prediction alone. This includes the measurement process, state dynamics, uncertainty calibration, counterfactual consistency, and policy-level consequences.^72^ Measurement validity concerns whether observed data adequately reflect the latent physiological state.^109^ Digital twins often infer an unobserved state $s_{t}$ from noisy and irregular observations:


$$o_{t}=g_{\phi }(s_{t})+\eta_{t},$$

where $o_{t}$ denotes observed clinical data at time t, $s_{t}$ denotes the latent physiological state, $g_{\phi }$ denotes the measurement model with parameters ϕ, and $\eta_{t}$ denotes measurement error. Validation should assess whether this mapping is reliable, including sensitivity to missingness, measurement bias, and temporally correlated errors.^110^

Dynamical adequacy concerns whether the transition model captures clinically relevant temporal behavior.^98^ In discrete time, this can be written as:


$$s_{t+1}=f_{\theta }(s_{t},a_{t})+\varepsilon_{t},$$

where $f_{\theta }$ is the state-transition model, $a_{t}$ is the clinical action, and $\varepsilon_{t}$ is the process noise. In continuous time, analogous models may be expressed as:


$$ds_{t}=b_{\theta }(s_{t},a_{t})dt+\Sigma_{\theta }(s_{t})dW_{t},$$

where $b_{\theta }$ is the drift function, $\Sigma_{\theta }$ is the diffusion term, and $dW_{t}$ is the stochastic variation. Validation should test whether the model reproduces relevant temporal signatures such as delays, recovery kinetics, oscillations, or regime-dependent responses.^99^

Calibration of predictive distributions is required because digital twins often support decisions under uncertainty. For a predictive quantile $q_{\alpha }$,


$$\mathrm{Pr}(Y\leq q_{\alpha }|T)\approx \alpha ,$$

where Y denotes the future outcome, $q_{\alpha }$ denotes the predicted α-quantile, and T denotes the digital twin. Calibration should hold not only overall, but also across clinically relevant subgroups, time horizons, and intervention regimes.^96^

This is essential because a model may discriminate well while producing risk estimates that are systematically overconfident or underconfident.^111^

Counterfactual consistency is needed when the twin is used to compare interventions.^10^^,^^17^ Such systems may estimate quantities such as:


$$E[Y|do(A=a),S_{t}=s],$$

where A denotes a clinical action, a denotes a specified action value, and $S_{t}=s$ denotes the current state. These quantities are meaningful only if the causal and mechanistic assumptions are coherent. Validation may therefore use negative controls, quasi-experimental variation,^112^ or regime-switching analyses to test whether the twin respects known causal constraints.^75^^,^^100^ Decision theory provides the natural link between prediction and action by evaluating forecasts according to expected utility, loss, preferences, and the consequences of alternative decisions.^113^

Then, policy validity concerns the consequences of decisions induced by the twin. Many intervention-oriented twins imply a decision rule,


$$\pi_{T}:s_{t}\mapsto a_{t},$$

where $\pi_{T}$ maps the inferred state to a recommended action. Its value may be written as:


$$V(\pi )=E_{\pi }[\sum_{t=0}^{T}\gamma^{t}r(s_{t},a_{t})],$$

where V(π) is the expected value of policy π, $r(s_{t},a_{t})$ the reward or clinical utility at time t, γa denotes discount factor, and T denotes the time horizon. Validation should therefore include off-policy evaluation when possible and prospective assessment when the twin influences real decisions.^97^ A policy that appears effective in silico may still destabilize care once deployed, especially if it changes clinician behavior or patient trajectories. Instrument-grade validation must anticipate these downstream effects (Table 3).

**Table 3.** Clinical exemplars of digital twins used as scientific instruments.

Type 1 diabetes: in silico trials + closed-loop “artificial pancreas” as an instrumented twin	References
In type 1 diabetes, digital-twin-like frameworks have been operationalized for years through validated in silico patient cohorts used to stress-test closed-loop insulin control algorithms. The UVA/Padova simulator enables controlled counterfactual experimentation (eg, meal patterns, sensor noise, delays) before clinical deployment, functioning as an instrument that mediates between mechanistic hypotheses and patient-indexed decision policies. Critically, this setting also makes drift tangible: small biases in sensing or update rules can remain “predictively acceptable” under historical policies while yielding unsafe control under policy shift, motivating instrument-grade validation beyond accuracy.	^114–117^
**ICU glycemic control: model predictive control as a continuously-updated “physiology twin”**	
In critical care, model-predictive-control (MPC) approaches to insulin titration instantiate an instrumented digital twin: the latent glucose–insulin state is inferred from sparse measurements, updated sequentially, and used to recommend actions under safety constraints. Randomized evaluations of MPC-based protocols show how “validation” must include safety under regime change (nutrition changes, steroids, vasopressors), not only point prediction. This domain provides a pragmatic template for auditing update frequency, robustness to missingness, and stability, core elements of instrument drift management.	^118^ ^,^ ^119^
**Ventricular tachycardia: patient-specific virtual heart to test counterfactual ablation strategies**	
In ventricular tachycardia, patient-specific computational heart models built from imaging have been used to identify arrhythmogenic substrates and to simulate alternative ablation targets before intervention. This is a canonical “scientific instrument” use-case: the twin supports counterfactual reasoning over interventions (lesion sets) that cannot be exhaustively tested in vivo, while making falsifiability operational (does the predicted inducibility/termination match EP outcomes?). The workflow also foregrounds scope: the twin is valid only for declared imaging quality, tissue characterization assumptions, and electrophysiologic regimes—naturally motivating an instrument validation statement.	^116^ ^,^ ^120^
**Persistent atrial fibrillation: computational modeling-guided ablation**	
Persistent atrial fibrillation highlights the action-versus-observation gap: observational correlations in electro-anatomic maps do not directly imply which lesions will terminate AF. Modeling-guided strategies explicitly encode intervention semantics by simulating the effect of candidate ablation patterns on patient-specific atrial dynamics. This setting maps tightly to instrument-grade evaluation: invariance under intervention, regime-switch testing, and calibration of uncertainty become as important as retrospective fit.	^121^ ^,^ ^122^
**Oncology (TNBC): MRI-based digital twins to optimize neoadjuvant chemotherapy regimens**	
In triple-negative breast cancer, MRI-informed digital twins have been constructed to forecast individual tumor response trajectories under neoadjuvant chemotherapy and to explore alternative regimens computationally. This is an “instrument” instantiation because the twin is dynamically anchored to patient-specific imaging, supports interventional counterfactuals (regimen choice/scheduling), and can be validated along multiple axes: measurement model adequacy (imaging-to-state mapping), dynamical adequacy (growth/response kinetics), and decision-level utility (regimen optimization under uncertainty).	^123^
**Neuro (drug-resistant focal epilepsy): “Virtual Epileptic Patient” to test counterfactual surgery/stimulation**	
In drug-resistant focal epilepsy, personalized “virtual brain” frameworks (eg, Bayesian/Virtual Epileptic Patient pipelines) fuse MRI/DTI-derived connectivity with SEEG/EEG to infer epileptogenic networks and to simulate counterfactual interventions such as virtual resections or stimulation strategies. Here, the instrument framing is natural: the twin mediates between mechanistic hypotheses (network excitability/propagation) and actionable decisions, while making uncertainty explicit and testable against seizure onset zones and surgical outcomes.	^124^ ^,^ ^125^

### The instrument validation statement: declaring epistemic scope

Because validation of a digital twin is inherently multi-dimensional, it cannot be summarized by a single performance metric.^72^^,^^85^ Instead, we propose that publications and regulatory submissions include an explicit instrument validation statement. In medical-device evaluation, credibility assessment frameworks similarly emphasize that the extent of verification and validation evidence should be commensurate with the model’s role in supporting a decision and with the consequences of that decision being wrong. Such a statement should delineate the epistemic scope of the twin by specifying the prediction horizons over which it has been evaluated, the classes of interventions it supports, the target populations to which it applies, the clinical outcomes it addresses, the uncertainty bounds it maintains, and the conditions under which its assumptions are known to fail.^86^

This statement can be operationalized as a concise structured paragraph rather than as a new performance metric. For example, an instrument validation statement could read:

This digital twin has been validated for [target population and care setting], using [data sources and measurement process], to support [specified decision or intervention classes] over [prediction horizon]. It provides [risk, trajectory, or policy output] with [uncertainty representation] and has demonstrated [calibration, discrimination, trajectory accuracy, updating stability, and/or policy evaluation] in [validation dataset or prospective setting]. It should not be used for [excluded populations, unsupported interventions, longer horizons, excessive missingness, or clinical regimes outside the validation data]. Outside this declared scope, outputs should be considered exploratory and interpreted with clinical judgment.

This practice mirrors the declaration of operating range and calibration for physical medical devices.^126^ Rather than claiming general validity, the instrument validation statement situates the digital twin within a defined domain of use, making explicit what it can and cannot be trusted to do. In doing so, it transforms validation from a retrospective performance claim into a prospective commitment, aligning scientific rigor with clinical accountability.

## Implications: how treating twins as instruments changes medicine

### Digital twins as experimental companions, not autonomous clinicians

Conceiving digital twins as scientific instruments fundamentally redefines their role in clinical practice.^127^^,^^128^ An instrument does not replace the clinician; it reshapes the epistemic space within which clinical reasoning unfolds.^95^^,^^129^ Imaging modalities, laboratory assays, and physiological monitors do not decide treatments autonomously, yet modern medicine would be inconceivable without them.^130^^,^^131^ Their value lies in their capacity to render certain aspects of reality observable, quantifiable, and comparable, while remaining embedded within a broader framework of expert interpretation. Digital twins, when treated as instruments, occupy an analogous position, albeit at a higher level of abstraction and integration.^72^

As experimental companions, digital twins support clinical and scientific exploration by enabling hypothesis testing, scenario comparison, and decision stress-testing under controlled assumptions.^2^^,^^132^^,^^133^ They allow clinicians to interrogate “*what-if*” questions that cannot be answered directly at the bedside, either because of ethical constraints, temporal irreversibility, or the impossibility of repeated experimentation on the same individual.^134^^,^^135^ Importantly, this exploratory function does not confer decision authority on the twin itself. Rather, it extends the clinician’s cognitive workspace, offering structured counterfactual evidence that must be weighed against contextual knowledge, patient preferences, and clinical judgment.

This framing contrasts sharply with narratives that portray digital twins as autonomous decision-makers or substitutes for clinical expertise.^84^ Such narratives obscure the epistemic status of the twin’s outputs, encouraging an unwarranted reification of simulated trajectories as actionable truths.^86^ By contrast, treating digital twins as experimental companions emphasizes their provisional and conditional nature.^81^^,^^85^ Their outputs are instrument readings, posterior distributions, simulated responses, and stress-tested scenarios, whose meaning emerges only through interpretation. In this sense, digital twins reinforce, rather than undermine, the centrality of human judgment by making explicit the assumptions, uncertainties, and tradeoffs that underlie clinical decisions.^127^

### Re-centering causal structure in AI-driven healthcare

Framing digital twins as instruments also re-centers of causal structure in AI-driven healthcare.^9^^,^^136^ Unlike purely predictive systems, which may achieve impressive performance while remaining agnostic to underlying mechanisms, an instrumented digital twin must commit to explicit causal assumptions in order to generate interpretable and actionable counterfactuals.^75^^,^^83^ These commitments concern not only which variables are included, but how they are related, which interventions are considered meaningful, and which sources of variation are treated as noise rather than signal.^100^

At the core of this work is the distinction between actions and exposures. In clinical contexts, not all variables are equally manipulable: treatments, dosages, and timing constitute deliberate interventions, whereas age, genetic background, or prior disease history are not subject to control.^97^ A digital twin that fails to encode this distinction risks conflating association with causation, thereby producing counterfactual recommendations that are incoherent or unsafe.^12^ The instrument framing requires that such distinctions be formalized, typically through explicit causal graphs or mechanistic equations, rather than left implicit in high-dimensional representations.^90^^,^^137^

This requirement extends naturally to the handling of confounding.^87^ In observational clinical data, treatment assignment is rarely random, and failure to address confounding by indication can lead to systematic misattribution of effects. A digital twin that purports to guide interventions must therefore make its confounding strategy explicit, whether through causal adjustment, structural modeling, or design-based validation. In doing so, the twin becomes interpretable in a scientifically meaningful sense. Interpretability here does not refer to post hoc explanations of feature importance, but to structural accountability: the ability to trace how assumptions about causality, control, and dependence give rise to specific counterfactual claims.^13^^,^^86^ By enforcing such accountability,^138^ the instrument framing offers a pathway to restore causal reasoning to the center of AI-enabled medicine.

### Regulatory science: auditing knowledge-producing systems

If a digital twin influences clinical decisions, directly or indirectly, it must be subject to regulatory scrutiny commensurate with its epistemic and clinical impact.^84^^,^^127^ Treating digital twins as instruments rather than algorithms has important implications for regulatory science. Traditional regulatory approaches to software often emphasize performance metrics and static validation, but these are insufficient for systems whose outputs depend on dynamic updating, counterfactual reasoning, and evolving clinical contexts.^139^ An instrumented digital twin is a knowledge-producing system, and its regulation must therefore address not only what it predicts, but also how it generates and updates its claims.^12^^,^^85^

Within this perspective, auditability becomes a central regulatory principle.^86^ Auditing a digital twin involves examining the structural commitments that underpin its operation, including its causal assumptions, mechanistic priors, and updating rules. It also requires assessing calibration over time and across contexts, ensuring that uncertainty estimates remain meaningful under distributional shift and that predictive intervals do not degrade silently as clinical practices evolve.^82^^,^^96^ Because digital twins may induce sequential decision policies, regulatory evaluation must also extend to policy-level effects, incorporating off-policy analyses and, where feasible, prospective validation in real-world settings.^97^

Crucially, auditability does not end at deployment. As with other medical instruments, post-market surveillance is essential to detect drift, loss of calibration, or unanticipated interactions with care pathways.^140^ Continuous monitoring and recalibration are not optional add-ons, but integral components of safe operation. By articulating regulatory requirements in terms of audits of assumptions, calibration, decision policies, and post-deployment behavior, the instrument framing provides a coherent template for governing digital twins without stifling innovation.^127^ It aligns regulatory oversight with the epistemic nature of the technology, ensuring that systems capable of shaping clinical knowledge are held accountable to standards appropriate to their role.

### Ethical accountability and responsibility when instruments fail

When digital twins influence clinical decisions, responsibility cannot be reduced to a simple opposition between developer and clinician.^84^^,^^138^ It is distributed across the socio-technical system that designs, validates, deploys, monitors, and interprets the twin.^141^ The key ethical issue is therefore to clarify, before deployment, who is responsible for which part of the system and under which conditions.

The instrument validation statement proposed here can support this clarification.^72^ By explicitly specifying the prediction horizons, classes of interventions, target populations, uncertainty bounds, and known conditions of failure for which a digital twin has been validated, the statement functions as an epistemic contract.^126^ Outside this domain, its output should be treated as exploratory or insufficiently validated.

Within this framework, developers are primarily responsible for the structural validity of the twin: the transparency and coherence of the transition model $f_{\theta }$, the measurement model $g_{\phi }$, the updating rule Π,^12^ and the causal assumptions supporting intervention-oriented outputs.^13^ They are also responsible for demonstrating calibration, robustness, falsifiability, and the absence of major failure modes such as systematic miscalibration, violation of invariance, or hidden confounding within the declared scope of use.^83^^,^^92^

Clinicians, by contrast, are responsible for contextual interpretation. They must assess whether the patient and decision context fall within the validated domain, integrate the twin’s outputs with clinical expertise, patient preferences, and external evidence, and exercise caution when uncertainty is high or assumptions are strained.^128^ When a twin is used outside its declared scope, responsibility shifts toward clinical discretion and institutional governance.

This distributed model avoids 2 risks: allowing developers to transfer responsibility to clinicians by deploying opaque systems with unclear limits, and holding clinicians responsible for failures that originate in the model structure or its validation.^142^ By making the scope of validity explicit, the instrument framing aligns accountability with control and makes responsibility more governable when digital twins influence care.^127^

## Conclusion

Medical digital twins are heterogeneous systems whose validation should depend on their intended use. For intervention-oriented digital twins, predictive performance is necessary but should not be the sole evidence of validity. When outputs are used to compare treatments, timings, dosages, or sequential policies, validation should also address calibration, uncertainty, updating stability, robustness under regime change, and decision-level consequences.

The scientific-instrument framing proposed here is not a universal definition of digital twins, but rather a pragmatic lens for systems that may influence clinical action. It helps define the scope within which twin outputs can be interpreted as conditional, model-mediated evidence. Medical digital twins should therefore be accompanied by explicit validation statements specifying their target population, prediction horizon, supported interventions, uncertainty bounds, and known failure conditions. Their safe use depends on matching the validation burden to the clinical claim being made.

## Data Availability

Not applicable.
